# Image Generation of Common Dermatological Diagnoses by Artificial Intelligence: Evaluation Study of the Potential for Education and Training Purposes

**DOI:** 10.2196/72371

**Published:** 2025-12-16

**Authors:** Sarah Kooper-Johnson, Subin Lim, Jumana Aldhalaan, Gabriela Cobos, Vijay Kodumudi, Daniel S Loo, Joshua Mervis, Madalyn Petrillo, Sarah N Robinson, Bichchau Michelle Nguyen

**Affiliations:** 1 Tufts Medical Center, 260 Tremont Street, Boston, MA, 02116, United States, 1 (617) 636-0156

**Keywords:** artificial intelligence, technology, morphology, education, diagnostic, image generation

## Abstract

**Background:**

The integration of artificial intelligence (AI) into dermatology holds promise for education and diagnostic purposes, particularly through image generation, which has not been well studied.

**Objective:**

This study aimed to assess whether AI image generation software can generate accurate images of classic dermatological conditions and whether they are recognizable as computer-generated.

**Methods:**

Images of 10 dermatological conditions were generated using DALLE-2 and DALLE-3 programs. These images were randomized among clinical photographs and distributed to dermatology residents and attending physicians. Participants were instructed to (1) identify AI-generated images and (2) provide their diagnosis.

**Results:**

AI-generated images were detected as computer-generated in 70.8% (85/120) of cases. Correct diagnoses were made based on all AI images 40.83% (49/120) of the time. This was significantly lower than the 72.0% (46/60) recognition rate for clinical photographs (*P*<.001). DALLE-2 images were diagnosed correctly less frequently (25.0%, 15/60) than DALLE-3 images (56.6%, 34/60; *P*<.001).

**Conclusions:**

AI-generated images of common dermatological conditions are becoming more accurate. This holds great implications for education but should be used with caution as further research is needed with more advanced, specific, and inclusive training data. Limitations include the use of AI image generators created by a single parent company as well as the use of a limited set of diagnoses.

## Introduction

The study and practice of dermatology is rooted in visual inspection and pattern recognition of classic disease morphology. Patient images in textbooks and archives aid in learning these patterns and developing differential diagnoses along with morphological descriptions. However, these images are not always immediately available and may have implications regarding patient privacy. Furthermore, historically, there has been significant limitations of availability of images depicting dermatological conditions in skin of color [1].

The use of artificial intelligence (AI) applications in dermatology has been described in many articles in which algorithms have neared or surpassed the diagnostic accuracy of clinicians when it comes to analyzing images [2]. New developments in AI have also reached mainstream headlines, as applications such as ChatGPT are able to perform well on dermatology boards style questions [3] and provide appropriate answers to patient questions [4] using complex neural networks. Their parent-company, OpenAI, also released a text-to-image generator in 2021 called DALL-E trained on text-images pairs sourced from across the internet. The autoregressive model produces images from scratch based on the text that the user inputs [5,6] .

This could have implications for the image-based field of dermatology, particularly in education. If AI can generate images of various conditions, these could be used alongside patient images as a quick reference tool and aid in the development of differential diagnoses. The aim of this study is to assess whether successive iterations of DALL-E can produce accurate images of classic dermatological conditions and whether these images can convincingly resemble clinical images or if they will be recognized as AI-generated.

## Methods

### Ethical Considerations

Given that this study did not include patient intervention or patient data, patient consent, de-identification, and privacy protections were not indicated. Additionally, this work was exempt from approval by the institutional review board..

### Study Overview

A list of 10 common dermatologic conditions (vitiligo, urticaria, rosacea, acne, milia, cellulitis, molluscum contagiosum, psoriasis, seborrheic dermatitis, and melasma) was generated by OpenAI’s DALLE-2 and DALLE-3. Clinical photographs, previously used as teaching Kodachromes, and deemed diagnostic of these 10 conditions by an attending physicians with >5 years of dermatology experience (BN) were also collected from an academic dermatologist with permission from patients for their use. The phrase “Please generate a photorealistic image of X on the Y” was used with each query where X was the dermatologic condition and Y was the location of the lesion or condition. For example, for acne, the phrase “please generate a photorealistic image of acne on the face” was inputted. During beta testing, it was found that using the diagnosis name as opposed to a morphological description yielded more accurate results.

Ten images from each generator were compiled and randomized among the 10 clinical photographs, a 50% ratio of images. A quiz containing all 30 of these images was administered to 2 second-year and 1 third-year dermatology resident, along with 3 attending physicians with >5 years of dermatology experience. Participants were instructed to (1) identify AI-generated images and (2) provide their diagnosis.

### Statistical Analysis

Two-sample z tests were used to determine if one image generator produced images that were more likely to be recognized as AI and to assess if there was a significant difference in the capability of these algorithms to produce recognizable and diagnosable images. These tests were also performed to see if residents or attending physicians were able to recognize AI images or come up with correct diagnoses at different rates. The results were also broken down by diagnosis category, for example inflammatory disorders and pigmentary disorder, and two-sample z tests were conducted to determine if the image generators were more recognizable as AI or more accurate with any particular category of diagnosis. Statistical significance level was set to α=.05. All statistical analyses were performed in Microsoft Excel.

## Results

**Figure 1.**
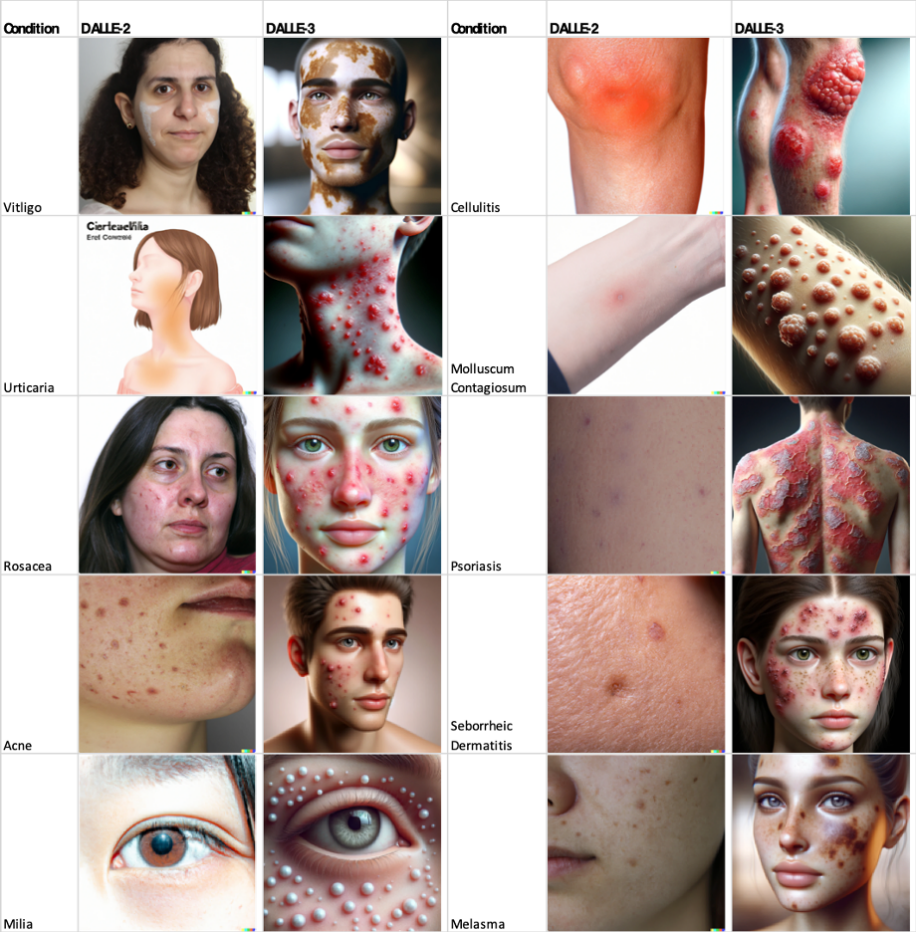
Conditions studied and images generated from DALLE-2 and DALLE-3 Software.

Upon subjective evaluation of AI-generated images, those generated by DALLE-2 exhibited a higher degree of photorealism compared to DALLE-3. However, these images tended to be non-specific and generally failed to accurately represent specific diagnoses. DALLE-3 images were more cartoonish in nature but showcased specific dermatological findings such as scale indicative of psoriasis. Of the 20 AI-generated images, none depicted individuals of older age, and only 2 images appeared to represent skin of color. These images can be found in Figure 1.

In the survey of dermatology residents and attending physicians, AI-generated images were detected as computer-generated in 70.8% (85/120) of cases. DALLE-2 images were recognized as AI-generated less often (41.66%, 25/60) than DALLE-3 images (100%, 60/60; *P*<.001). Clinical photographs were mistakenly perceived as AI-generated in only 1.66% (1/60) of instances. Residents recognized all AI images more often than attending physicians (75% [45/60] vs 66.67% [40/60]; *P*=.0224) and recognized DALLE-2 images as AI generated more often than attending physicians (50% [15/30] vs 33.33% [10/30]; *P*=.001). Both groups recognized DALLE-3 images as AI generated 100% of the time.

In terms of producing recognizable images, correct diagnoses were made based on all AI images 40.83% (49/120) of the time. This was significantly lower than the 71.67% (43/60) recognition rate for clinical photographs (*P*<.001). When comparing image generators, DALLE-2 images were diagnosed correctly less frequently, at 25.0% (15/60), compared with DALLE-3 images, which were diagnosed correctly 56.67% (34/60) of the time (*P*<.001). Attending physicians were able to correctly identify DALLE-2 images more often than residents (36.67% [11/30] vs 20% [6/30]; *P*=.001). The rate of correct diagnosis was the same for residents and attending physicians for DALLE-3 images (56.67%, 34/60). DALLE-2 images representing urticaria, psoriasis, and seborrheic dermatitis were misclassified the most often in this set. The DALLE-3 image representing cellulitis was misclassified the most often in this set. The full analysis results are shown in Table 1.

**Table 1.** Quiz results broken down by respondent type describing how often respondents recognized images as artificial intelligence (AI) generated and how often the correct diagnosis was given.

Respondents	Clinical images	All AI images	DALLE-2 images	DALLE-3 images	*P* value between DALLE-2 and DALLE-3
Percentage recognized as AI					
All respondents	1.66 (1/60)	70.83 (85/120)	41.66 (25/60)	100 (60/60)	<.001
Resident respondents	0 (0/30)	75 (45/60)	50 (15/30)	100 (30/30)	<.001
Attending physician respondents	3.33 (1/30)	66.67 (40/60)	33.33 (10/30)	100 (30/30)	<.001
*P* value between resident and attending physician responses	.747	.022	.001	1	
Percentage of given correct diagnosis					
All respondents	71.67 (43/60)	40.80 (49/120)	25 (15/60)	56.67 (34/60)	<.001
Resident respondents	80 (24/30)	38.33 (23/60)	20 (6/30)	56.67 (34/60)	<.001
Attending physician respondents	63.33 (19/30)	46.67 (28/60)	36.67 (11/30)	56.67 (34/60)	<.001
*P* value between resident and attending physician responses	.001	.002	.001	1	

When broken down by disease category, AI-generated images of infectious diseases from all programs were recognized as AI the most often at 83.33% (20/24). Across all categories, DALLE-3 images were recognized significantly more often than DALLE-2 images (*P*<.001 for each). Infectious etiologies also had low rates of correct diagnosis, being correctly labeled only 16.67% (2/12) of the time for both DALLE-2 and DALLE-3 images. Otherwise, DALLE-3 images were given the correct diagnosis more often than DALLE-2 images across categories, with pigmentary disorders being the most accurate at 91.67% (11/12) recognition. The full analysis results are shown in Table 2.

**Table 2.** Quiz results broken down by disease category describing often respondents recognized images as artificial intelligence (AI) generated and how often the correct diagnosis was given.

Disease category	Inflammatory	Infectious	Pigmentary	Other
Percentage recognized as AI (All AI programs)	65 (39/60)	83.33 (20/24)	70.83 (17/24)	66.67 (8/12)
Percentage given correct diagnosis (clinical images)	80 (24/30)	66.67 (8/12)	83.33 (10/12)	16.67 (1/6)
Percentage given correct diagnosis (all AI programs)	36.67 (22/60)	16.67 (4/24)	70.83 (17/24)	41.67 (5/12)
*P* value between all AI and clinical images for correct diagnosis	<.001	<.001	.019	.012
Percentage recognized as AI (DALLE-2)	30 (9/30)	66.67 (8/12)	41.67 (5/12)	50 (3/6)
Percentage recognized as AI (DALLE-3)	100 (30/30)	100 (12/12)	100 (12/12)	100 (6/6)
*P* value between DALLE-2 and DALLE-3 for AI recognition	<.001	<.001	<.001	<.001
Percentage given correct diagnosis (DALLE-2)	26.67 (8/30)	16.67 (2/12)	41.67 (5/12)	0 (0/6)
Percentage given correct diagnosis (DALLE-3)	46.67 (14/30)	16.67 (2/12)	91.67 (11/12)	83.33 (5/6)
*P* value between DALLE-2 and DALLE-3 for correct diagnosis	<.001	>.99	<.001	<.001

## Discussion

The objective of this work was to determine if the AI image generator DALL-E is able to produce realistic and accurate depictions of common dermatologic conditions. As the field of AI continues to advance, its integration into dermatology holds promise for educational and diagnostic purposes. However, this study reveals nuances in the accuracy and recognition of AI-generated images compared to clinical photographs.

One notable aspect of the results is the varying recognition rates of AI-generated images, with DALLE-3 images being recognized as computer-generated more often than DALLE-2 images. This was seen across disease categories and across levels of training. This could be due to controversy surrounding these tools and implications regarding images that were too realistic such that later iterations of the software were made to generate images that were more cartoonish in nature. It is pertinent, however, that residents were better able to recognize computer-generated images, particularly the more photorealistic ones. This may indicate that the younger generation is more in tune with this technology and may have implications for its future use.

Nevertheless, it was seen that the later software, DALLE-3, produced more accurate images, with the correct diagnosis being assigned more often than with DALLE-3 images. DALLE-3 performed particularly well when generating images of pigmentary disorders. This indicates that the programs could be receiving more training data on this topic over the course of time and indicates promise for future programs. However, attending physicians were more likely to give the correct diagnosis for DALLE-2 generated images, suggesting that there may be more subtle signs present in these images that indicate the correct diagnosis. Neither program generated images that were correctly diagnosed as often as clinical images, indicating room for improvement on this front as well.

Finally, skin of color was represented only twice in the dataset. Although skin of color images could be generated upon instruction, it is evident that this is not the default setting for these tools. Considering that patients of color already face barriers to dermatological diagnoses and treatment [7], it is imperative that training sets of images for any type of algorithm are inclusive and representative of a diverse population.

Limitations of this study include the use of AI image generators created by a single parent company as well as the use of a limited set of diagnoses. Further research is needed in this area with more advanced and specific training data. As with any new technology, careful research should be conducted as it improves to ensure that if and when it is utilized for patient care, it upholds standards of safety, accuracy, and equity.

## Abbreviations

AI: artificial intelligence
